# Impact of SiO_2_ doping on the structure and oil–water separation properties of a PVDF membrane: insights from molecular dynamics simulation

**DOI:** 10.1039/d4ra03807j

**Published:** 2024-07-31

**Authors:** Yi Liu, Jing Zhang, Jiale Li, Yuxing Zhao, Ming Zhang

**Affiliations:** a School of Chemistry and Chemical Engineering, Tianjin University of Technology Tianjin 300384 China zm2404@tjut.edu.cn; b Center of Membrane Materials and Engineering Technology, Tianjin University of Technology Tianjin 300384 China

## Abstract

Hybrid inorganic particles combined with polymers are widely used to modify the properties of polymer membranes. However, the mechanism by which particles affect membranes remains unclear. This study investigates SiO_2_-hybridized PVDF membranes through molecular dynamic simulation, focusing on the interaction between SiO_2_ clusters and PVDF chains. It examines the impact of varying SiO_2_ concentrations (3.5 wt%, 6.8 wt%, 9.9 wt%, 12.8 wt%, and 15.5 wt%) on membrane stability and structure. The results indicate that adding SiO_2_ can inhibit PVDF chain mobility in the membrane with minimal effect on fractional free volume (FFV), except for altering interactions between PVDF–PVDF, PVDF–SiO_2_, and SiO_2_–SiO_2_, thereby affecting the structure of hybrid membranes. The adsorption and diffusion behavior of water and oil molecules on these membranes were also studied. It was observed that the adsorption energy and diffusion coefficient initially increase and then decrease with increasing SiO_2_ concentration, reaching an optimum between 6.8 wt% and 12.8 wt%. This phenomenon is attributed to the ability of optimal SiO_2_ concentrations to create hydrophilic channels in PVDF membranes, enhancing water affinity and reducing oil affinity. Consequently, water permeation through the hybrid membrane is promoted, improving the efficiency of oil/water separation compared to pure PVDF membranes. This research contributes to understanding the function of adding inorganic particles to polymer membranes and provides insights for designing advanced functional hybrid membranes.

## Abstract 2

Separation mechanism of SiO_2_-doped PVDF (polyvinylidene fluoride) membrane for oil–water mixtures.

## Graphical abstract

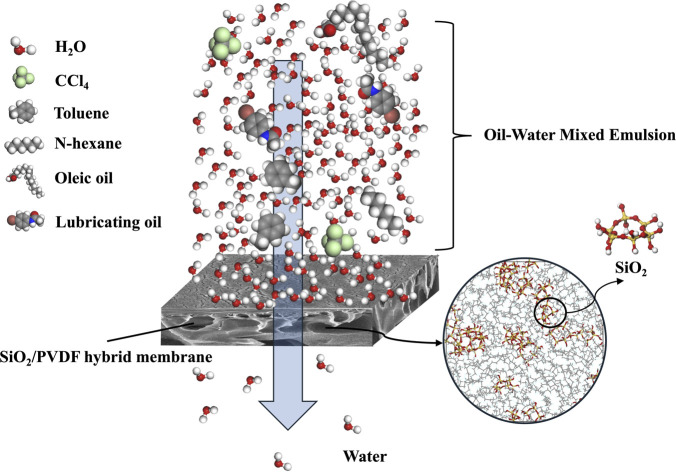

## 1 Introduction

With the increasing demand for industrial production, the generation of numerous oily wastewaters has caused significant damage to the environment and human health. Consequently, addressing the challenge of water pollution through effective oil–water separation has become imperative. Common methods for separating oil–water mixtures include centrifugation,^1,2^ adsorption purification,^3,4^ membrane separation,^5,6^ and others. Among these, membrane separation technology has garnered considerable attention due to its advantages of low energy consumption, simple operation, strong selectivity, environmental friendliness, and high separation accuracy.^7^ Polymer membranes, notably polyvinylidene fluoride (PVDF),^8–10^ polyether sulfone (PES)^11–13^ and polypropylene (PP)^14–16^ are widely utilized in practical applications.

The PVDF membrane, in particular, has emerged as the most extensively employed membrane owing to its commendable mechanical properties, ease of cleaning, and reusability.^17^ It has been selected as an efficient liquid separation membrane for emulsified water-in-oil/oil-in-water solutions in many studies. Hai's lab^18^ has prepared a superhydrophobic and superoleophilic PVDF membrane using the phase inversion method, achieving a high flux of 8800 L m^−2^ h^−1^. Nie and coworkers^19^ employed a simple one-step method to achieve hydrophilic modification of electrospun PVDF nanofiber membranes for oil–water separation, consistently achieving separation efficiencies exceeding 99.50%.

To enhance membrane performance, particularly its anti-fouling properties,^20^ various methods have been adopted, such as surfactant modification,^21,22^ grafting,^23,24^ blending^25,26^ and loading nanoparticles onto the membrane. Common inorganic particles suitable for hybridization with PVDF membranes include TiO_2_,^27–29^ Al_2_O_3_,^30–32^ and SiO_2_.^33–35^ Among these, SiO_2_ stands out as the most widely used due to its mild reactivity and well-known chemical properties. Xu *et al.*^36^ developed a superhydrophilic tea polyphenols/silica composite coating, resulting in an ultrahigh water flux of 15 353 L m^−2^ h^−1^. Additionally, the modified membrane achieved highly efficient separation of oil/water emulsions (above 96%). Xu's lab^37^ prepared a superhydrophobic/superoleophilic SiO_2_/PVDF membrane, exhibiting stable fluxes for petroleum ether/water, *n*-hexane/water, and *n*-heptane/water at 3886 ± 140, 3551 ± 146, and 3763 ± 57 L m^−2^ h^−1^, respectively, with separation efficiencies consistently above 99.7%. Wen *et al.*^38^ prepared cotton fiber@SiO_2_ superhydrophobic fabric can be used for the separation of oil/water mixtures with a separation efficiency of 98%.

The particles can obviously improve the performance of the PVDF membrane; however, the interaction among the particles, polymer membrane, and oil/water molecules is still not clear. To unravel the effect of nanoparticle on the membrane properties, some simulation works have been adopted. Liang *et al.*^39^ designed three kinds of modified SiO_2_ nanoparticles grafted with hydrophilic chains, hydrophobic chains, and mixed hydrophilic and hydrophobic chains to detach adsorbed oil droplet on silica surface. Similarly, Bai's lab^40^ investigated the effects of the size of silica particles on the diffusion behavior of water in SiO_2_/PVDF hybrid membranes. Even though more simulation work^41–43^ has been done, the interaction mechanism between particles and membrane, as well as the surface interaction between oil/water and hybrid membrane, is still not clear. Furthermore, how the nanoparticles affect the separation of oil–water emulations remains uncertain.

In this study, a model of a SiO_2_/PVDF hybrid membrane with various oil and water molecules was constructed. The aggregation of SiO_2_ nanoclusters with different concentrations in the PVDF membrane was investigated, and the interaction among SiO_2_, PVDF, and oil/water molecules was analyzed. Based on the analysis of the interaction, the effect of SiO_2_ on the structure of PVDF membranes and separation performance for oil–water emulation were studied. This study unveils the adsorption and diffusion mechanisms of oil and water with SiO_2_/PVDF hybrid membrane at the molecular level, providing a theoretical foundation for the design and development of high-performance oil/water separation membranes.

## 2 Model and methodology

### 2.1. Models and molecules

The MD simulations were carried out using Materials Studio 7.0 software,^44^ the SiO_2_ clusters was built as follows: initially, the α-SiO_2__quartz was imported, then a spherical shape was chosen, and the radius was set to 3 Å using the Build Nanocluster function. The resulting SiO_2_ clusters were then saturated with hydrogen. Next, hydrogen atoms directly connected to the Si atoms were screened out and replaced with oxygen atoms. Finally, the atoms were hydrogen saturated again.^45^ The purpose of this process is to ensure that the oxygen atoms directly connected to the Si atoms are bonded and saturated, and to protonate them to form Si–OH groups, thus avoiding subsequent bonding deficiencies that could result in unsuccessful model optimization.

The PVDF single chain, consisting of 40 difluoroethylene repeating units, was constructed using the Forcite module.^46^ SiO_2_ clusters were then randomly distributed within the PVDF membrane to form a hybrid SiO_2_/PVDF model. The concentration of SiO_2_ varied from 3.5 wt% to 15.5 wt%, while the initial density of the PVDF membrane was set to 1.7 g cm^−3^. The box length along the *a* and *b* directions was fixed at 50 Å.

**Fig. 1:**
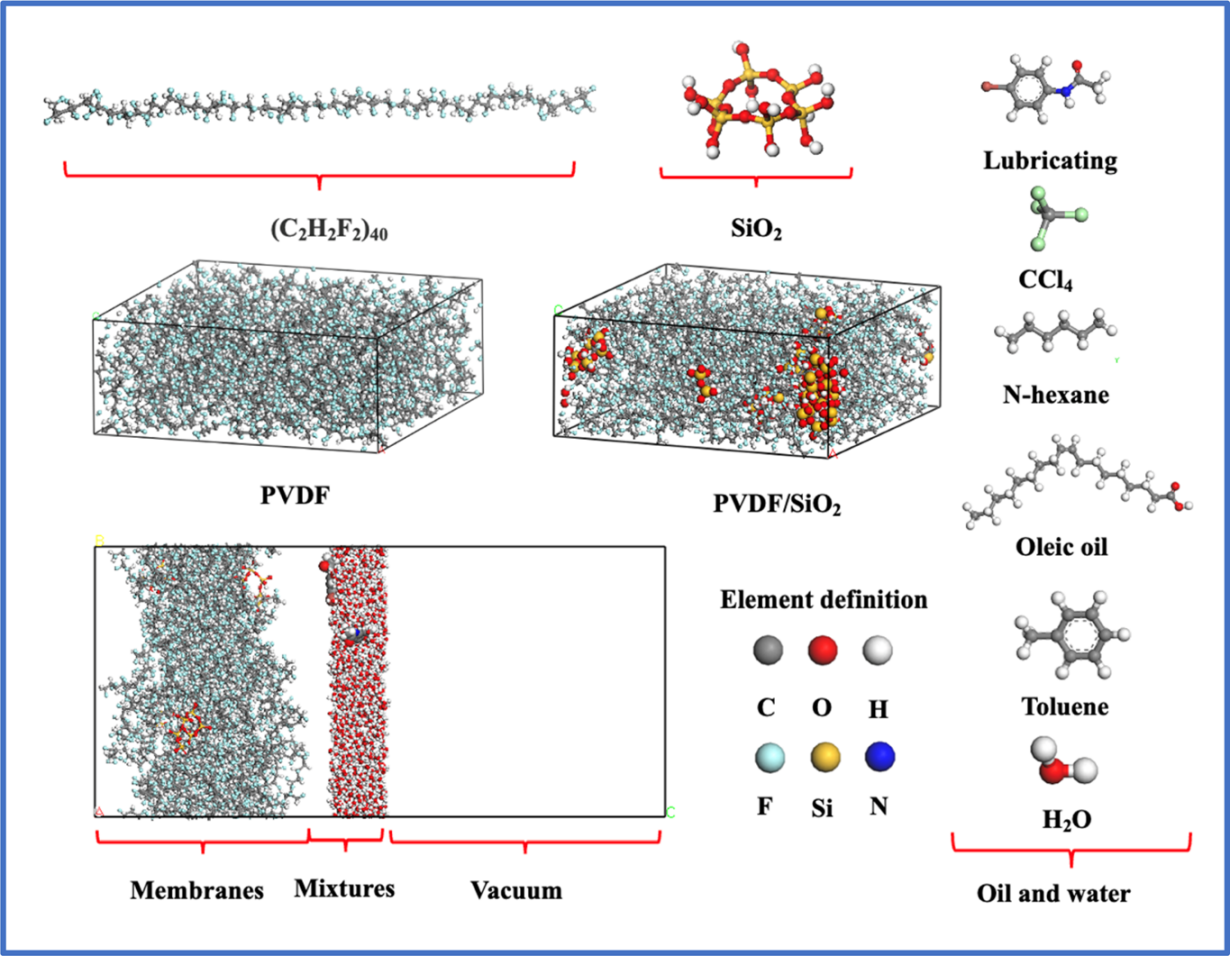
Models of the PVDF, SiO_2_/PVDF and oil molecules.

The oil–water mixture was placed on the surface of SiO_2_/PVDF. Carbon tetrachloride, *n*-hexane, toluene, oleic acid, and lubricating oil were chosen as representatives of oil-in-water emulsions based on their molecular weights and different functional groups. The density of the oil–water mixing layer is 1 × 10^3^ kg m^−3^, and the molar ratio content of oil in oil-in-water emulsions is approximately 3 wt% ± 0.5. The effect of periodic boundaries on the adsorption results is eliminated by creating a vacuum layer with a thickness of about 50 Å on the membrane layer. The models used in this work were shown in Fig. 1.

### 2.2. Simulation details

#### 2.2.1 Energy optimization

For all models, energy minimization was conducted using the Smart minimizer method,^47^ transitioning from the steepest descent method^48^ to the conjugate gradient method,^49^ and then to the quasi-Newton^50^ as the energy derivative decreased to expedite computation. Next, an annealing procedure was performed where the systems were heated from 298 to 598 K at intervals of 50 K and then cooled at intervals of 20 K. The process involved 5000 iterations, with optimizations repeated several times to rectify issues such as interatomic distances or overlaps, thereby generating the initial structure for kinetic simulations. The simulation was carried out entirely within the Forcite module, utilizing the condensed-phase optimized molecular potentials atomistic simulation studied (COMPASS)^51^ force field consistently throughout the modeling process.

#### 2.2.2 Dynamic simulation

Geometry optimization was initially conducted to relax the system, succeeded by dynamic simulations aimed at acquiring the equilibrium configurations of both the pure PVDF membrane and the SiO_2_/PVDF hybrid membrane system. Subsequently, the models obtained post-annealing optimization underwent a 200 ps NPT optimization to fully relax the membranes, in which the magnitude of pressure is set to 3 × 10^−4^ GPa. This was followed by a series of 1 ns NVT tether simulations, repeated iteratively until the density of each system reached stabilization. Finally, a 1 ns equilibrium structural treatment was executed utilizing the NVT ensemble at a temperature of 298 K, employing a time step of 1 fs.

The Anderson thermostat and barostat^52^ were used to maintain the temperature and pressure for all dynamic simulations. A group-based cut-off distance of 12.5 Å was used to avoid interactions between the non-bonded van der Waals and electrostatic forces. The long-range electrostatic interactions were evaluated by the Ewald summation method. The coordinates of the system were collected every 10 000 steps. Trajectory frames were captured during the production run and the data from the final 1000 ps was used for analysis. The well-validated COMPASS force field was selected for all MD simulations because it has been widely used for accurate prediction of the structural and thermophysical properties of polymer molecules under various conditions.

#### 2.2.3 Diffusion coefficient

The permeation properties of oil and water molecules are closely related to their motility, and the mean square displacement (MSD) is one of the important parameters to characterize the structural changes in the membrane chain and their diffusion within the PVDF membrane, which is calculated using eqn (1):

1 MSD(*t*) = 〈|*r*_*i*_(*t*) − *r*_*i*_(0)|^2^〉

where *r*_*i*_(0) and *r*_*i*_(*t*) denote the position of atom *i* at the initial and *t* moments, respectively. *N* is tabulated as the number of atoms containing diffusive motion in the whole system.2
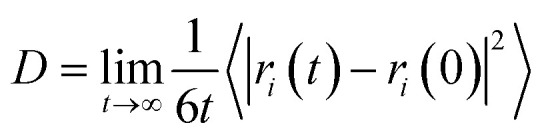

3 *D* = 〈MSD〉/(6*t*)

Diffusion coefficient *D* is a physical quantity that expresses the diffusion speed of atoms, and it is a simple method to analyze the diffusion behavior of atoms in the membrane from a microscopic point of view through molecular simulation, and the diffusion coefficient *D* can be obtained by the calculation of eqn (2), and the relationship between MSD and diffusion coefficient *D* in the MS calculation is shown in eqn (3).

#### 2.2.4 Fractional free volume

The membrane harbors a substantial pore space, denoted as the fractional free volume (FFV), which significantly influences both the macroscopic morphology of the membrane and the analytical outcomes. The characterization of the size and distribution of the free volume within polymer membranes is facilitated by computational tools such as MS software employing the Atom Volume & Surface tool. The FFV of both PVDF membranes and SiO_2_/PVDF hybrid membranes is determined utilizing the following equation:4
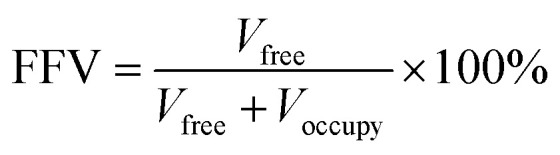
where *V*_free_ and *V*_occupy_ represent the volumes of free and occupied space within the membrane, respectively:

Furthermore, the ratio of large and small holes of various diameters is derived through the subsequent formula:5
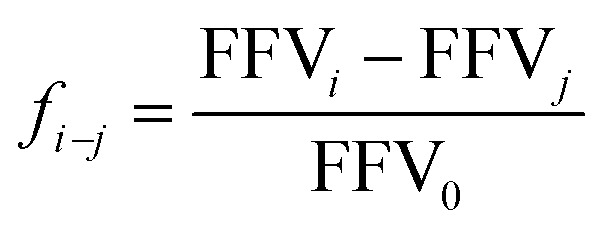
where FFV_*i*_, FFV_*j*_, and FFV_0_ correspond to the FFVs when the probe radius is *i*, *j*, and 0 Å, respectively.

#### 2.2.5 Adsorption energy

An in-depth understanding of adsorption mechanisms can provide insights into how molecules approach a surface and interact effectively. The adsorption energy serves as a critical tool for determining whether small molecules have undergone effective adsorption. The calculations were performed using the Forcite module in MS, maintaining accuracy at the Fine level. This energy is computed using the formula:

6 *E*_ads_ = −(*E*_total_ − *E*_membrane_ − *E*_small_)

where *E*_total_ represents the single point energy of the entire model after adsorption stabilization, *E*_membrane_ denotes the single point energy of the PVDF or SiO_2_/PVDF membrane remaining after the removal of oil molecules, and *E*_small_ denotes the single point energy of the small molecules remaining after the removal of the membrane structure. The resulting energy, after their subtraction, represents the adsorption energy corresponding to each small molecule.

## 3 Results and discussion

### 3.1. The influence of SiO_2_ on the stability of the PVDF membrane

#### 3.1.1 The mobility of the PVDF chain in the membrane

The mobility of polymer chains is closely associated with the transport properties of hybrid membranes. The presence of SiO_2_ is considered to play a key role in the mobility of PVDF chains within the membrane.^52^ The values of mean square displacement (MSD) of PVDF molecules before and after SiO_2_ doping were investigated; a larger slope of the MSD curve indicates greater mobility of the PVDF chain.

**Fig. 2:**
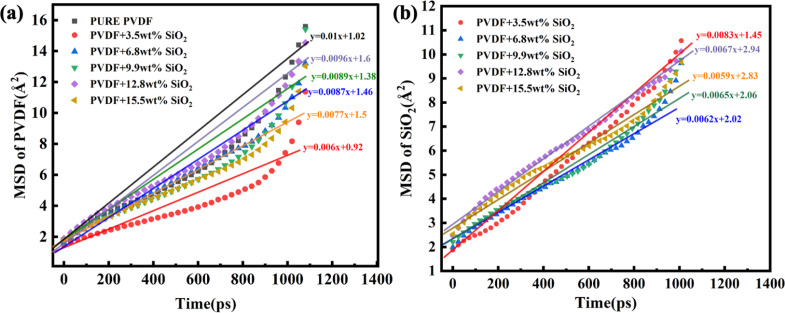
MSD of PVDF chains (a) and SiO_2_ nanoclusters (b).

The MSD values of PVDF molecules in membranes with and without SiO_2_ are shown in Fig. 2a. It is evident that the slope of the curve for the pure PVDF membrane is 0.01. As SiO_2_ nanoparticle doping increases, the slope value exhibits a slight decrease from 0.01 with 0 wt% addition of SiO_2_ to 0.006 with 3.5 wt% addition, followed by an increase with further SiO_2_ addition, reaching 0.0087 at 6.8% and 0.0089 at 9.9 wt%. Subsequently, the slope decreases again to 0.0077 with 15.5 wt% SiO_2_ addition. Notably, the slope value for the 9.9 wt% SiO_2_ addition is 0.0089, which closely resembles that of the pure PVDF membrane.

These results indicate that the addition of SiO_2_ can inhibit the mobility of PVDF chains in the membranes. The slope of the pure PVDF membrane is highest at 0.01. At low concentrations of SiO_2_, the slight decrease can be attributed to the distribution of SiO_2_ among PVDF chains. The binding interaction between SiO_2_ clusters and chains decreases the mobility of PVDF. However, with increasing SiO_2_ addition, the SiO_2_ tends to aggregate in the membrane, weakening the interaction between SiO_2_ and PVDF. Consequently, the mobility of PVDF exhibits a slight increase. With further SiO_2_ addition, the SiO_2_ clusters occupy more space, pushing the PVDF chains closer together, leading to a notable decrease in PVDF mobility at 15.5 wt% addition, reaching 0.0077.

The aggregation of SiO_2_ clusters with various additions in the membrane has been analyzed using the MSD of SiO_2_, as shown in Fig. 2b. It is clear that with the increase in SiO_2_, the slope of the curve exhibits an obvious decrease. The slope for 3.5 wt% SiO_2_ is 0.0083, but for the 9.9 wt% addition of SiO_2_, it decreases to 0.0065. With the concentration of SiO_2_ increasing to 15.5 wt%, it further decreases to 0.0059. This indicates that the movement of SiO_2_ clusters weakens with the increasing addition of SiO_2_ in the membrane.

**Fig. 3:**
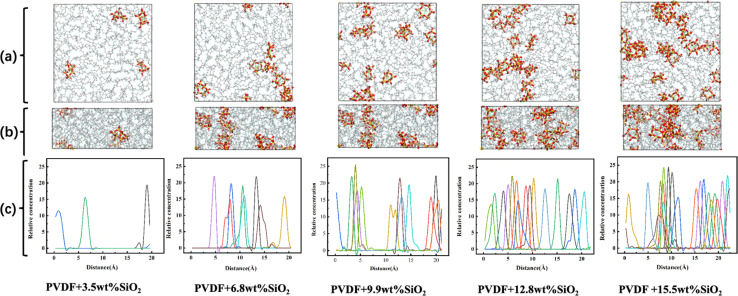
The distribution of SiO_2_ nanoparticles in hybrid membranes along the *Z*-axis. (a) Top view, (b) side view, (c) relative concentration profiles (each color curve represents a different SiO_2_ cluster in the membrane).

The aggregation of SiO_2_ at various concentrations in the hybrid membrane is also illustrated in Fig. 3. As depicted in the top and side views in Fig. 3a and b, it is evident that with the addition of 3.5 wt%, the four SiO_2_ clusters were uniformly deposited within the membrane. However, with an increase in SiO_2_ concentration, slight aggregation of clusters is observed at 6.8 wt% and 9.9 wt%. Moreover, with further increases in SiO_2_ concentration to 12.8 wt% and 15.5 wt%, larger aggregations appear in the membrane. The distribution of SiO_2_ clusters with different concentrations along the *Z*-axis in the membrane is also shown in Fig. 3c, where each color curve represents a different SiO_2_ cluster in the membrane. It is observed that the peaks exhibit increasing overlap with higher concentrations of SiO_2_, particularly at 12.8 wt% and 15.5 wt%, indicating that the different SiO_2_ clusters aggregate at higher concentrations. This observation is consistent with the MSD curves of PVDF chains in the hybrid membranes as depicted in Fig. 2.

#### 3.1.2 Interaction energy of the PVDF chains and SiO_2_ clusters

The motivation and conformation of PVDF are influenced by interactions among PVDF chains and SiO_2_ clusters within the membrane. Three types of interaction energy were examined for the hybrid membrane: the interaction energy between PVDF chains (denoted as *E*_PVDF–PVDF_, eqn (7)), the interaction energy between PVDF and SiO_2_ (denoted as *E*_PVDF–SiO_2__, eqn (8)), and the interaction energy between SiO_2_ particles (denoted as *E*_SiO_2_–SiO_2__, eqn (9)).

Eqn (7) is represented as:7
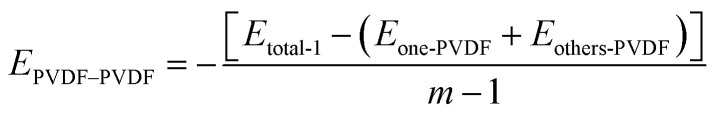
Here, *E*_total-1_ represents the total energy of the membrane with all SiO_2_ clusters removed. *E*_one-PVDF_ and *E*_others-PVDF_ represent the single-point energy of one PVDF chain and the rest of the PVDF chains in the membrane without any SiO_2_ clusters, respectively, where *m* is the number of PVDF chains in the membrane.

Eqn (8) is represented as:8
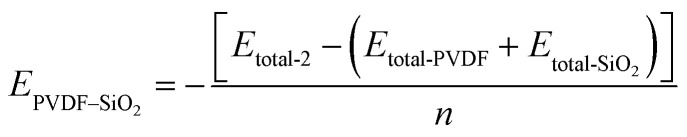
Here, *E*_total-2_ represents the total energy of the PVDF membrane with the addition of all SiO_2_ clusters. *E*_total-PVDF_ and *E*_total-SiO_2__ represent the single-point energy of all PVDF chains and all SiO_2_ clusters in the membrane, respectively, where *n* is the number of SiO_2_ clusters in the membrane.

Eqn (9) is represented as:9
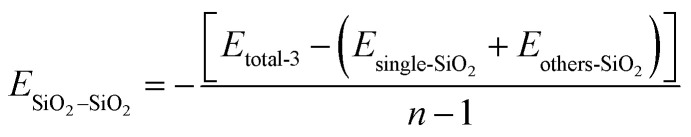
Here, *E*_total-3_ represents the total energy of all SiO_2_ clusters. *E*_single-SiO_2__ and *E*_others-SiO_2__ represent the energy of one SiO_2_ clusters and the remaining SiO_2_ clusters, respectively, where *n* is the number of SiO_2_ clusters in the membrane.

**Fig. 4:**
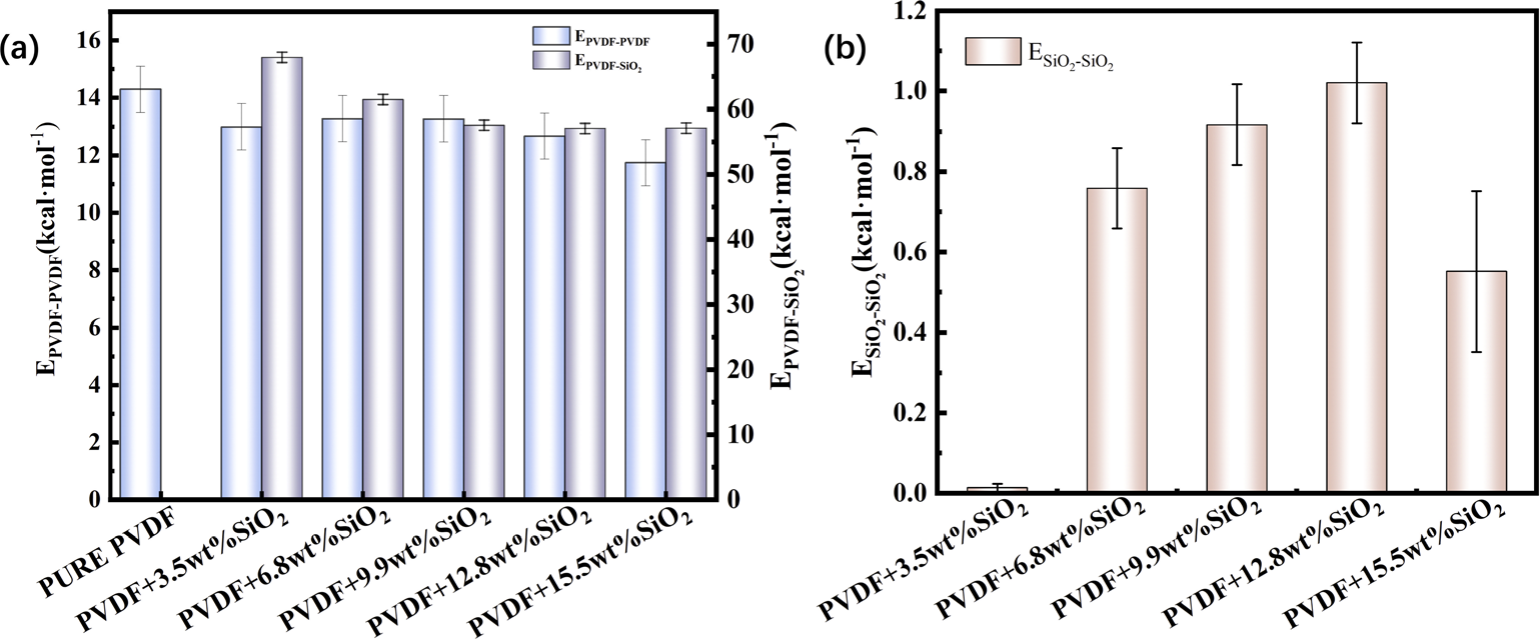
Interaction energy of PVDF–PVDF, PVDF–SiO_2_ (a) and SiO_2_–SiO_2_ (b).

The three kinds of interaction within the hybrid membrane were depicted in Fig. 4. As shown in Fig. 4a, it is evident that the interaction between PVDF chains in the pure PVDF membrane is highest, at 14.3 kcal mol^−1^. With the increasing concentration of SiO_2_, the *E*_PVDF–PVDF_ exhibits a slight decrease to 13.0 kcal mol^−1^ for 3.5 wt%, then increases again to 13.3 kcal mol^−1^ for 6.8 wt% and 13.4 kcal mol^−1^ for 9.9 wt%, subsequently decreasing to 12.7 kcal mol^−1^ for 12.8 wt% addition of SiO_2_, and further to 11.7 kcal mol^−1^ for 15.5 wt% of SiO_2_. This changing trend suggests that the addition of SiO_2_ decreases the interaction between PVDF chains. This can be explained by the addition of SiO_2_ distributing among the PVDF chains, thereby weakening the interaction between PVDF chains, which is also consistence with the mobility of PVDF chain within the membrane as shown in Fig. 2.

The fluctuation of *E*_PVDF–PVDF_ with various concentration of SiO_2_ also can be contributed to the distribution and aggregation of SiO_2_ clusters in the membrane. As shown in Fig. 4b, at an addition concentration of 3.5 wt% SiO_2_, the interaction between SiO_2_ clusters is at its lowest, only 0.01 kcal mol^−1^, because the small amount of SiO_2_ is distributed far apart from each other, more than 10 Å (as seen in Fig. 2), resulting in almost no interaction between them. With an increase in the concentration of SiO_2_, the *E*_SiO_2_–SiO_2__ exhibits a noticeable increase. For the concentration of 12.8 wt% SiO_2_, the *E*_SiO_2_–SiO_2__ is about 1.0 kcal mol^−1^, indicating that SiO_2_ clusters form spurious aggregation. This can also be observed in Fig. 2b.

The aggregation of SiO_2_ clusters also affects the interaction between PVDF chains and SiO_2_. As shown in Fig. 4a, the *E*_PVDF–SiO_2__ exhibits a noticeable decrease from 68.0 kcal mol^−1^ with a 3.5 wt% concentration of SiO_2_ to 57.2 kcal mol^−1^ with a 15.5 wt% concentration of SiO_2_. This occurs because, for the same number of SiO_2_ clusters, the aggregation of SiO_2_ reduces the total contact area between SiO_2_ clusters and PVDF chains compared to dispersed SiO_2_.

#### 3.1.3 The conformation of PVDF chains

**Fig. 5:**
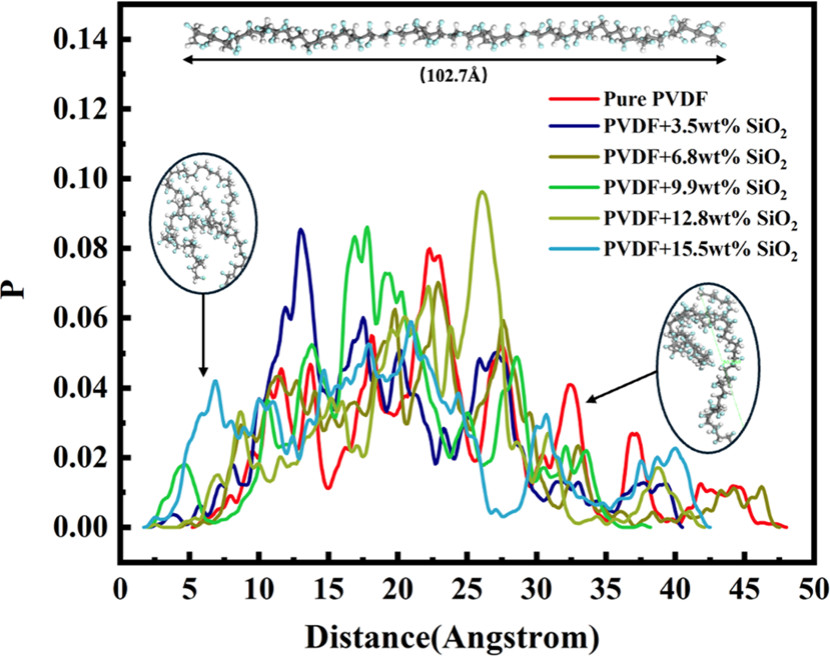
Distribution of the distance between the head and the tail of the PVDF chains.

The addition of SiO_2_ not only affects the mobility of PVDF chains, but also influences their conformation. The distance between the head atom and the tail atom of the PVDF chain was investigated in this work. The distribution of this distance for 20 PVDF chains in the membranes is shown in Fig. 5. The linear conformation of the PVDF chain, as depicted in the upper inset of Fig. 5, has a chain length of about 102 Å. However, PVDF chains in the membrane exhibit various distortions and bending, resulting in chain lengths distributed among 3–45 Å, as shown in the insets in Fig. 5. The length distribution for membranes with and without SiO_2_ addition is focused on the range of 7–45 Å, suggesting a potentially stable conformation. In membranes without SiO_2_ addition, the head–tail length distribution ranges from 3–47 Å. With different concentrations of SiO_2_ addition, slight differences are observed in the distribution curve: 3–38 Å after adding SiO_2_ nanoparticles with a concentration of 3.5 wt%, 3–35 Å after adding SiO_2_ nanoparticles with a concentration of 9.9 wt%, and 3–42 Å after adding SiO_2_ clusters with a concentration of 15.5 wt%. This implies that the addition of SiO_2_ can affect the conformation of PVDF chains, potentially intensifying their folding and causing a more closely distributed head–tail length. This could be attributed to the compression between PVDF chains after adding SiO_2_ clusters to the membrane.

### 3.2. Effect of SiO_2_ on the structure of the PVDF membrane

#### 3.2.1 The pore distribution of the SiO_2_/PVDF membrane

**Fig. 6:**
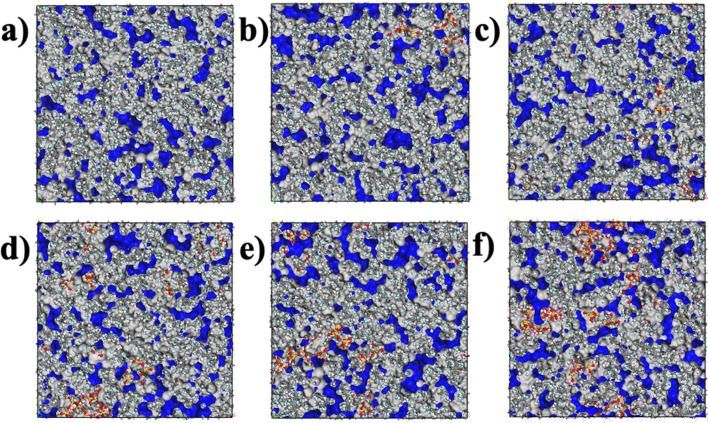
Free volume inside the SiO_2_/PVDF membranes: (a) pure PVDF, (b) PVDF + 3.5 wt% SiO_2_, (c) PVDF + 6.8 wt% SiO_2_, (d) PVDF + 9.9 wt% SiO_2_, (e) PVDF + 12.8 wt% SiO_2_, (f) PVDF + 15.5 wt% SiO_2_. The blue denotes the area of free volume. *Via* the hardsphere probe method, the probe radius (*R*_p_) = 0.5.

The separation property of the membrane correlates with the pore size and distribution within it. The fractional free volume (FFV) of the membranes can be used to characterize the pore structure and distribution. The FFV, using a probe of 0.5 Å radius, of membranes both without and with various additions of SiO_2_, is shown in Fig. 6. The blue-marked area represents the pores formed in the membrane. From Fig. 6, it can be observed that the pore size and distribution in membranes with and without SiO_2_ exhibit only slight differences. For membranes without SiO_2_, the pores are small, but with the addition of SiO_2_, there is a slight increase in pore size, and they become interconnected. This suggests that the presence of SiO_2_ can create hydrophilic channels in the PVDF membrane, which enhances water flux and is beneficial for improving overall performance.

**Fig. 7:**
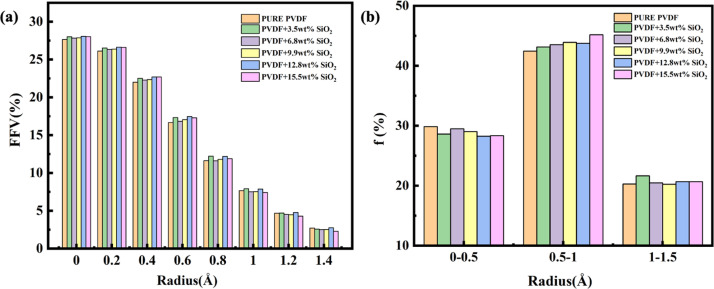
FFV of membranes (a) and ratio of large and small holes of different diameters (b).

The FFV with different radius probes for various membranes is also presented in Fig. 7. As depicted, the pore distributions are similar, even for pure PVDF membranes or SiO_2_ hybrid PVDF membranes. Furthermore, it is evident that the overall porosity of all membranes, with or without SiO_2_, is approximately 28% volume/volume (v/v), with a notably higher proportion of small pores compared to larger ones. For instance, pores under 0.2 Å radius constitute more than 25%, whereas pores over 1.2 Å radius are less than 5%.

Additionally, as illustrated in Fig. 7b, medium-sized pores, ranging between 0.5–1.0 Å (radius), account for 45% of the total pore volume. Pores with a probe radius of 0–0.5 Å constitute less than 30%, while those with a probe radius of 1.0–1.5 Å comprise approximately 20%. This variation in pore size distribution is consistent across various membranes. The pore distribution analysis of membranes with and without SiO_2_ suggests that the addition of SiO_2_ has a minimal impact on pore size and distribution within the membrane.

#### 3.2.2 Diffusion coefficient of water molecules in membranes

**Fig. 8:**
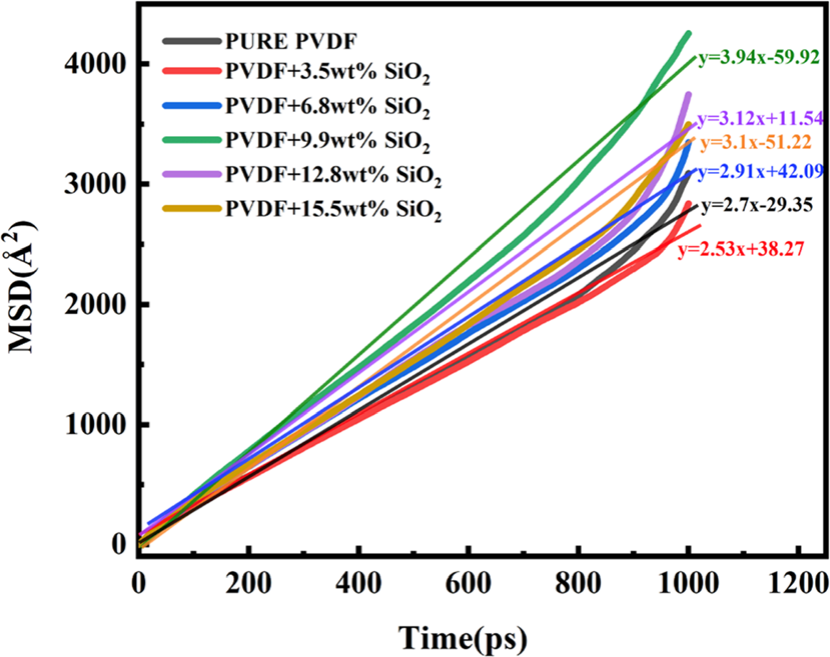
Change in diffusion coefficient of water.

The MSD curves illustrating water molecule diffusion in different membranes are depicted in Fig. 8. The slope of the MSD curve reflects the diffusion performance of water molecules within the membrane. As shown in Fig. 8, it is evident that the slope of the pure PVDF membrane is 2.70. With the addition of 3.5 wt% SiO_2_, the slope decreases to 2.53. Subsequently, with an increase in SiO_2_ concentration, the slope increases to 2.91 for 6.8 wt%, 3.94 for 9.9 wt%, decreases to 3.12 for 12.8 wt%, and 3.10 for 15.5 wt%. Correspondingly, the diffusion coefficient of water in the membranes follows a similar trend as the MSD (shown in Table 1), with the pure PVDF membrane exhibiting approximately 0.45 × 10^−6^ cm^2^ s^−1^. As SiO_2_ concentration increases, water molecule diffusion coefficient initially decreases and then increases. Except for the membrane with 3.5 wt% SiO_2_ addition, where the diffusion coefficient (*D* value) is 0.42 × 10^−6^ cm^2^ s^−1^, generally, hybrid membranes exhibit higher water molecule diffusion compared to pure PVDF membranes. Notably, the addition of 9.9 wt% SiO_2_ yields the highest diffusion of 0.66 × 10^−6^ cm^2^ s^−1^ among all membranes.

**Table 1:** Diffusion coefficients of water in the PVDF membrane and SiO_2_/PVDF hybrid membranes

Membranes	Diffusion coefficient (1 × 10^−6^ cm^2^ s^−1^)
Pure PVDF	0.45
PVDF + 3.5 wt% SiO_2_	0.42
PVDF + 6.8 wt% SiO_2_	0.49
PVDF + 9.9 wt% SiO_2_	0.66
PVDF + 12.8 wt% SiO_2_	0.52
PVDF + 15.5 wt% SiO_2_	0.51

The variation in water diffusion coefficient can be attributed to the mobility of PVDF chains and the hydrophilicity of SiO_2_ clusters within the membrane. At lower concentrations, such as 3.5 wt%, SiO_2_ distributes well within the membrane, limiting the mobility of PVDF chains (as shown in Fig. 2). The presence of SiO_2_ between PVDF chains may obstruct some membrane channels, thereby reducing the formation of diffusion paths and subsequently decreasing the chance of water molecule passage through membrane pores, resulting in a smaller diffusion coefficient. As the SiO_2_ addition increases, SiO_2_ clusters can form hydrophilic channels within the membrane. This increase in the membrane's hydrophilicity further promotes the transfer of water molecules within the membrane, with the maximum diffusion reaching 0.66 × 10^−6^ cm^2^ s^−1^ at 9.9 wt%. However, with further SiO_2_ increases, SiO_2_ nanoclusters aggregation exerts a repulsive effect on water transfer, leading to a slight decrease in the diffusion coefficient (*D* value). These results suggest that the impact of SiO_2_ addition on water diffusion through the membrane is concentration-dependent. In this study, the optimal formulation is found to be 9.9 wt% addition of SiO_2_.

### 3.3. Effect of SiO_2_ on membrane separation of oil-in-water emulsions

In many works,^53,54^ the addition of SiO_2_ has been shown to enhance the separation efficiency of PVDF membranes. However, the impact of SiO_2_ on the separation mechanism for various oil–water emulsions remains unclear. This section investigates the adsorption of water and typical oils (carbon tetrachloride, lubricating oil, oleic acid, hexane, toluene) on the hybrid membrane, as well as the diffusion of oil–water emulsion through the membrane.

#### 3.3.1 Adsorption of water and oil molecules on the membrane

**Fig. 9:**
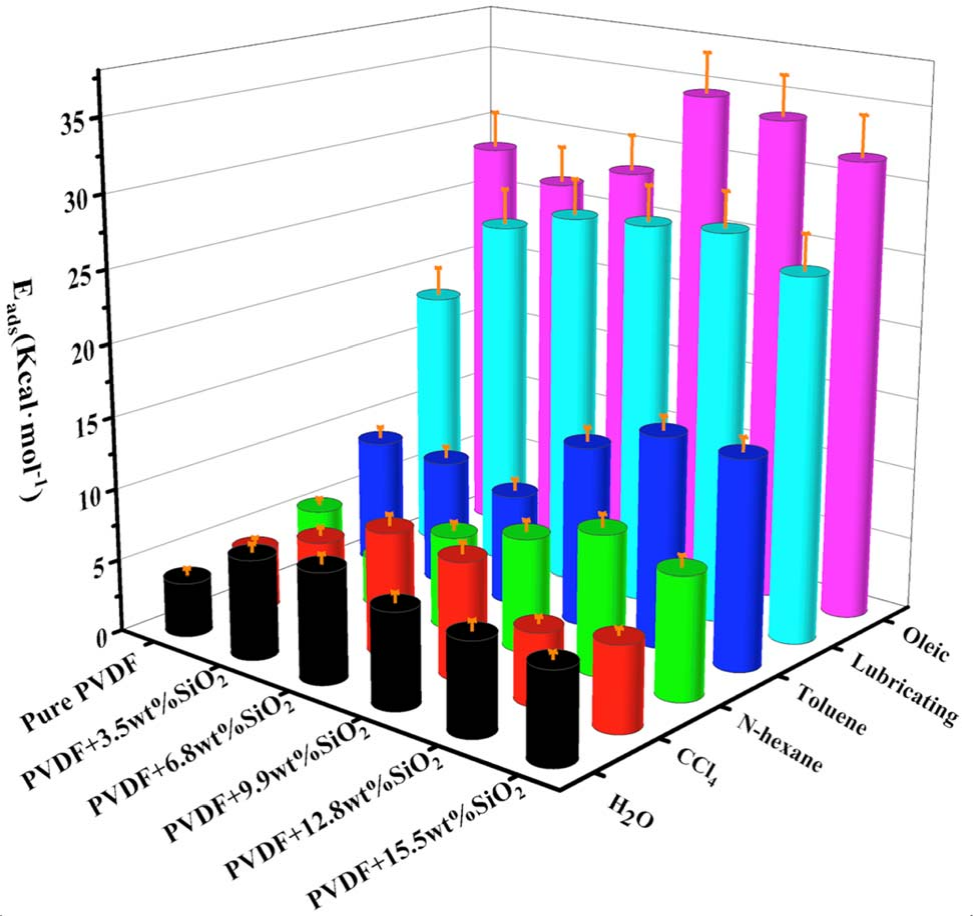
Adsorption energy of water and oil molecules on the membranes.

The adsorption energy of different molecules on the membranes is depicted in Fig. 9. It is evident that the adsorption energy (*E*_ads_) of various oils on the pure PVDF membrane increases with the carbon atom number. However, the *E*_ads_ of oils on the hybrid membranes with varying concentrations of SiO_2_ nanoclusters show an overall trend of initially increasing and then decreasing with the increase in SiO_2_ concentration. Larger oil molecules such as lubricating (C_8_H_8_BrNO) and oleic (C_18_H_34_O_2_) exhibit higher *E*_ads_ values, exceeding 20 kcal mol^−1^, which are notably higher than those of other molecules. This is attributed to the relatively larger molecular weight of lubricating and oleic. The higher adsorption energy for different oil molecules is observed within the SiO_2_ concentration range of 6.8 wt% −12.8 wt%.

The relative adsorption energy of the hybrid membrane for water or oils can influence the separation of oil–water emulsions. The ratio of *E*_ads_ (water) to *E*_ads_ (oil) is presented in Table 2. A higher value of *E*_ads_ (water)/*E*_ads_ (oil) indicates better adsorption for water and poorer adsorption for oil. As depicted in Table 2, the addition of SiO_2_ enhances the adsorption of water for all oil–water emulsions. For instance, for the H_2_O/*n*-hexane emulsion, the membrane with 3.5 wt% SiO_2_ demonstrates good performance. Similarly, for the emulsions of H_2_O/toluene, H_2_O/lubricating, and H_2_O/oleic, the membrane with 6.8 wt% SiO_2_ exhibits better performance. This suggests that for different oil–water emulsions, the optimal hybrid membrane requires varying additions of SiO_2_ nanoclusters.

**Table 2:** Ratio of *E*_ads_(water)/*E*_ads_(oil) on SiO_2_/PVDF membranes

	H_2_O/CCl_4_	H_2_O/*n*-hexane	H_2_O/toluene	H_2_O/lubricating	H_2_O/oleic
Pure PVDF	0.90	0.70	0.43	0.21	0.13
PVDF + 3.5% SiO_2_	1.09	**2.07**	0.80	0.28	0.26
PVDF + 6.8% SiO_2_	0.90	1.19	**1.00**	**0.29**	**0.27**
PVDF + 9.9% SiO_2_	0.82	0.85	0.53	0.25	0.19
PVDF + 12.8% SiO_2_	**1.29**	0.67	0.44	0.24	0.19
PVDF + 15.5% SiO_2_	1.07	0.75	0.43	0.25	0.19

**Fig. 10:**
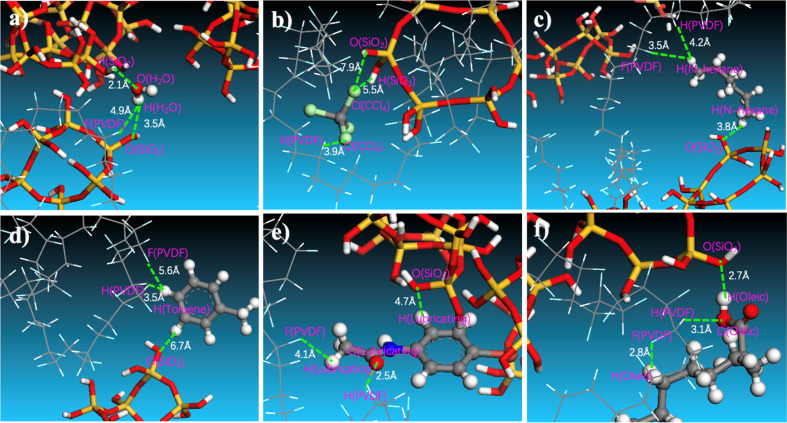
Snapshots of water and oil molecules (a) H_2_O, (b) CCl_4_, (c) *n*-hexane, (d) toluene, (e) lubricating oil, and (f) oleic acid permeating through the SiO_2_/PVDF hybrid membrane containing 9.9 wt% SiO_2_. The PVDF chain is depicted in a linear model, SiO_2_ particles are represented in stick form, and water and oil molecules are shown using a ball and stick model. The color scheme assigns red to oxygen (O), grey to carbon (C), white to hydrogen (H), yellow to silicon (Si), blue to nitrogen (N), green to chlorine (Cl), and cyan to fluorine (F) atoms.

The effect of SiO_2_ on the separation of oil/water emulsions can be attributed to interactions between oil molecules and the membrane. A typical snapshot in Fig. 10 illustrates the relative positions of water or oil molecules within the SiO_2_ (9.9 wt%) hybrid PVDF membrane. Specifically, the distance between the hydrogen atom of H_2_O and the oxygen of hydrated SiO_2_ is approximately 3.5 Å. In contrast, the distances between the hydrogen atoms of *n*-hexane (Fig. 10c), toluene (Fig. 10d), and lubricating oil (Fig. 10e) and the oxygen of hydrated SiO_2_ are elongated to 3.8 Å, 6.7 Å, and 4.7 Å, respectively.

Conversely, the distance between the oxygen atom of H_2_O and the hydrogen atom of PVDF is 4.9 Å (Fig. 10a), which is greater than the distance between the chlorine atom of CCl_4_ (Fig. 10b) and the hydrogen atom of PVDF (3.9 Å), as well as the distances between the oxygen atom of lubricating oil (Fig. 10e) and the hydrogen atom of PVDF (2.5 Å), and between the oxygen atom of oleic acid (Fig. 10f) and the hydrogen atom of PVDF (3.1 Å). These indicate that oil molecules have a certain affinity with the pure PVDF chain. However, the addition of SiO_2_ increases the membrane's affinity for water and reduces its affinity for oils. This enhancement promotes water permeation through the membrane and improves the efficiency of oil/water separation in PVDF membranes.

#### 3.3.2 Diffusion of oil–water emulsion through the hybrid membrane

**Fig. 11:**
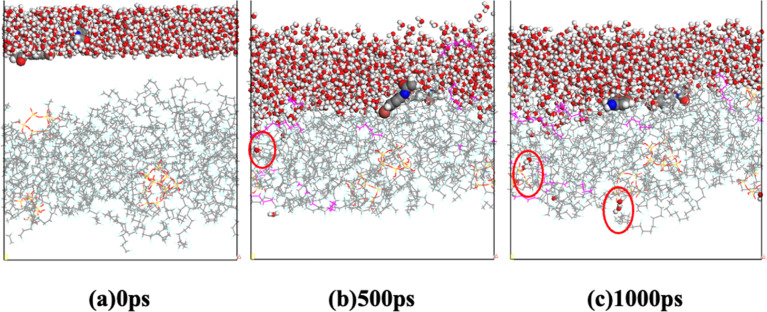
Diffusion process of lubricating-water on the PVDF membrane with a 3.5 wt% addition of SiO_2_ for different time intervals: (a) 0 ps, (b) 500 ps, and (c) 1000 ps. Water is represented using a ball and stick model, membranes are depicted using a line model, and oil is represented using a CPK model.

The interaction between the oils/water and the membranes influences the diffusion of molecules within the membrane. Fig. 11 illustrates the diffusion of lubricating-water emulsion through the PVDF membrane with 3.5 wt% SiO_2_ hybridization at different time points (0 ps, 500 ps, 1000 ps). It is evident that the lubricating molecules are obstructed on one side of the membrane, while the water molecules gradually diffuse through the membrane pores to the other side. This phenomenon is advantageous for the separation of oil–water emulsion.

## 4 Conclusion

This study investigated the impact of SiO_2_ on PVDF membrane structure and performance through molecular dynamic simulation. Results indicate SiO_2_ addition initially increases then decreases motility and interaction energy of PVDF chains, affecting membrane structure. Low concentration SiO_2_ addition weakens PVDF chain interaction, enhancing chain mobility. While pore size is minimally affected by hybridization, diffusion coefficient slightly increases with 9.9 wt% SiO_2_. SiO_2_ incorporation significantly influences adsorption diffusion of oil and water molecules, with aggregation affecting diffusion patterns. Variations in SiO_2_ concentration show different effects on oil–water emulsion separation, aiding high-performance membrane design. Overall, the study offers insights into nanoparticle–polymer chain interaction for membrane optimization in oil–water separation applications.

## Data availability

Correspondence and requests for materials and data should be addressed to Ming Zhang.

## Author contributions

Yi Liu developed the concept, collected information, designed and executed the simulations, analyzed the data, and wrote the manuscript. Jing Zhang and Jiale Li assisted in simulations and conducted data analysis. Yuxing Zhao conducted preliminary simulations during the concept development stage. Ming Zhang supervised the research, revised the manuscript, and discussed the results.

## Conflicts of interest

The authors declare no competing financial interests.
